# Transcobalamin 2 orchestrates monocyte proliferation and TLR4-driven inflammation in systemic lupus erythematosus via folate one-carbon metabolism

**DOI:** 10.3389/fimmu.2024.1339680

**Published:** 2024-05-31

**Authors:** Baoyi Liu, Ang Li, Yi Liu, Xinzhu Zhou, Jingkai Xu, Xianbo Zuo, Ke Xue, Yong Cui

**Affiliations:** ^1^ Department of Dermatology, China-Japan Friendship Hospital, Beijing, China; ^2^ Graduate School, Peking Union Medical College and Chinese Academy of Medical Sciences, Beijing, China; ^3^ Graduate School, Capital Medical University, Beijing, China; ^4^ Department of Dermatology, Peking University China-Japan Friendship School of Clinical Medicine, Beijing, China; ^5^ Department of Pharmacy, China-Japan Friendship Hospital, Beijing, China

**Keywords:** SLE, transcobalamin II, folate one-carbon metabolism, S-adenosylmethionine, TLR4

## Abstract

**Background:**

SLE is a complex autoimmune disease with deleterious effects on various organs. Accumulating evidence has shown abnormal vitamin B12 and one-carbon flux contribute to immune dysfunction. Transcobalamin II (TCN2) belongs to the vitamin B12-binding protein family responsible for the cellular uptake of vitamin B12. The role of TCN2 in SLE is still unclear.

**Methods:**

We collected clinical information and blood from 51 patients with SLE and 28 healthy controls. RNA sequencing analysis, qPCR, and western blot confirmed the alteration of TCN2 in disease monocytes. The correlation between TCN2 expression and clinical features and serological abnormalities was analyzed. TCN2 heterozygous knockout THP1 cells were used to explore the effects of TCN2 dysfunction on monocytes. CCK-8 assay and EdU staining were used to detect cell proliferation. ELISA was conducted to assess vitamin B12, glutathione, and cytokines changes. UHPLC-MRM-MS/MS was used to detect changes in the intermediates of the one-carbon cycle. Flow cytometry is used to detect cell cycle, ROS, mitoROS, and CD14 changes.

**Results:**

Elevated TCN2 in monocytes was correlated positively with disease progression and specific tissue injuries. Using CD14+ monocytes and TCN2 genetically modified THP1 cell lines, we found that the TCN2 was induced by LPS in serum from SLE patients. TCN2 heterozygous knockout inhibited cellular vitamin B12 uptake and one-carbon metabolism, leading to cell proliferation arrest and decreased Toll-like receptor 4 (TLR4)-mediated CCL2 release. Methionine cycle metabolites, s-adenosylmethionine and homocysteine, rescued these effects, whereas folate treatment proved to be ineffective. Folate deficiency also failed to replicate the impact of TCN2 downregulation on THP1 inflammatory response.

**Conclusion:**

Our study elucidated the unique involvement of TCN2-driven one-carbon flux on SLE-associated monocyte behavior. Increased TCN2 may promote disease progression and tissue damage by enhancing one-carbon flux, fostering monocyte proliferation, and exacerbating TLR4 mediated inflammatory responses. The inhibition of TCN2 may be a promising therapeutic approach to ameliorate SLE.

## 1 Introduction

Systemic lupus erythematosus (SLE) is an autoimmune disease with unknown causes. It is characterized by dysregulation of both adaptive and innate immune pathways. Monocytes, which are essential components of the innate immune system, originate from hematopoietic stem and progenitor cells (HSPC) in the bone marrow and express CD14 and CD16 (1). The role of pro-inflammatory monocytes in autoimmune diseases is well-documented (2, 3). Recent research has highlighted their significant contribution to SLE progression. Mononucleosis is a distinct characteristic observed in mice susceptible to SLE (4). In human studies, monocytes are found to be increased in the blood and kidneys of SLE patients (5). These monocytes serve as a source of cytokines that contribute to the pathogenesis of SLE and are associated with kidney damage (6).

Folate one-carbon metabolism is a universal metabolic process in cells. It comprises the folate cycle and the methionine cycle, which generate nucleotides, methyl groups, and glutathione. These components are necessary for cell proliferation and redox defense. The process has been extensively studied in tumors and is considered a promising therapeutic target (7, 8). Recently, several studies highlighted the role of one-carbon flux in inflammation and immune regulation (9–11). Enhanced one-carbon metabolism promotes pro-inflammatory macrophage polarization (12). Methotrexate (MTX) can induce macrophages to acquire immune tolerance by blocking one-carbon metabolism (13). MTX is a structural analog of folic acid (FA). It inhibits dihydrofolate reductase (DHFR) and thymidylate synthase (TS) in the folate cycle and methionine adenosyltransferase (MAT) in the methionine sulfate cycle (14, 15). It is been widely used to treat autoimmune diseases such as SLE, implied that inhibiting one-carbon metabolism may be a viable strategy for treating autoimmune diseases.

Transcobalamin II (TCN2) belongs to the vitamin B12-binding protein family. Its function is to aid in the cellular uptake of vitamin B12, making it the most reliable biomarker for active and available vitamin B12 (16). Vitamin B12 serves as a cofactor for methionine synthase (MTR) and methionine synthase reductase (MTRR), which play a crucial role in catalyzing the remethylation of homocysteine to methionine in one-carbon metabolism (10, 16). Studies have reported elevated TCN2 levels and decreased vitamin B12 levels in the blood of SLE patients (17–19). However, the role of TCN2 in SLE remains largely unknown.

In this study, we observed increased TCN2 expression in monocytes from SLE patients, which correlated positively with disease activity. TCN2 in monocytes was upregulated by SLE patient serum and lipopolysaccharide (LPS). The function of TCN2 in monocytes was investigated by constructing TCN2 heterozygous knockout THP1 cells (TCN2-KO, targeting position is EXON2) using CRISPR technology. TCN2-KO inhibited cell proliferation and reduced LPS-induced inflammatory cytokine release by inhibiting one-carbon metabolism. Our study sheds light on the potential implications of TCN2 in the pathogenesis of SLE and highlights the intricate involvement of folate one-carbon metabolism in monocyte-mediated inflammatory processes.

## 2 Materials and methods

### 2.1 Patient samples

A total of 51 peripheral blood samples were collected from patients diagnosed with SLE, along with 28 peripheral blood samples from age-matched healthy individuals, following the receipt of informed consent. The SLEDAI-2K scale was used for disease activity score, ≥ 7 was classified as active SLE (20). Detailed patient information is listed in **Supplementary Table 1**. This study obtained approval from the regional ethics committee and adhered to the principles outlined in the Declaration of Helsinki. Peripheral blood samples were collected using EDTA-containing anticoagulation blood vessels, centrifuged at 1500 rpm, centrifuged for 5 min, and the upper serum was stored at -80°C; meanwhile, the precipitated layer was used to isolated peripheral blood mononuclear cells (PBMC) or CD14+ monocytes.

### 2.2 Cell collection and treatment

PBMCs were isolated by density gradient centrifugation utilizing Ficoll-Paque™ (GE Healthcare, USA). Briefly, blood samples were diluted with sterile PBS and gently layered atop the Ficoll-Paque, then centrifuged at 1800 rpm for 20 minutes with the brake off at 20°C, then PBMC were collected from the interface. CD14+ monocytes were isolated using StraightFrom^®^ Whole Blood CD14 MicroBeads (Miltenyi Biotec, USA). Blood samples were incubated with CD14 magnetic beads for 15 minutes and then separated using a MACS column placed within a magnetic field. After two rinses with separation solution, CD14+ cells were collected with eluent buffer. The human monocytic cell line THP1 cells were obtained from the National Biomedical Experimental Cell Resource Bank (China). TCN2 heterozygous knockout THP1 cell (TCN2-KO cell) was purchased from UBIGENE (China). The knockout of TCN2 was achieved by CRISPR technology. Since the coding region sequence of exon 1 of TCN2 is too short, the gRNA targeting position was shifted to the most anterior region of exon 2. Exon 2 is well-organized among all coding transcripts, and targeting it affects all coding transcripts. Therefore, we deleted the fragment on exon 2 using double gRNA guidance. We confirmed the genotype of TCN2 KO cells through sanger sequencing. The results showed that of the two alleles, one gene was successfully knocked out and the other was a multiple of three genotypes (**Supplementary Figure 1**). Cells were cultured in RPMI-1640 containing 10% fetal bovine serum and 1% penicillin-streptomycin solution at 37°C with 5% CO2. LPS (L4391) and S-adenosylmethionine (SAM, A7007) were purchased from Sigma (USA), N-acetylcysteine (NAC, HY-B0215), Hcy (homocysteine, HY-W010347) were purchased from MCE (China), and FA (IF0180) was purchased from Solarbio (China).

To establish normal, FA-deficient, or FA-supplemented conditions, THP-1 cells were cultured for 3 days in either normal RPMI-1640 medium (supplemented with 1 μg/ml FA) or FA-free RPMI-1640 medium (27016021, Thermo Fisher Scientific, USA) or by adding FA (with final concentration of 10 μg/ml FA) to normal RPMI-1640 medium (IF0180, Solarbio, China).

### 2.3 Cell proliferation and cycle assays

Cells were seeded into 96-well plates (5000 cells/well) and cultured for 0 to 5 days. To assess cell proliferation, 10 µl of CCK-8 reagent (Yeasen, China) was added to each well containing 100 µl of medium. The plates were then incubated at 37°C for 2 hours, after which the optical density (OD) was measured at 450 nm using a microplate reader (Thermo Scientific, USA). The BeyoClick™ EdU Cell Proliferation Kit (Beyotime, China) was utilized for EdU staining according to the manufacturer’s instructions. In brief, cells were incubated with a 10 μM EdU solution for 6 hours, then centrifugated at 300g for 5 minutes to collect the cells. Subsequently, the cells were fixed at room temperature in 4% paraformaldehyde for 15 minutes, washed with PBS three times, permeabilized with 0.3% Triton X-100 in PBS for 10 minutes, and incubated in Click Additive Solution in the dark for 30 minutes. Cell nuclei were stained with Hoechst for 10 minutes. For cell cycle analysis, cells (1×10^6^ cells/well) were incubated with DNA labeling solution (CYT-PIR-25, Cytognos, Spain) in the dark for 10 minutes at room temperature. Subsequently, cell cycle data were acquired using a flow cytometer (BD Calibur, USA) operating at a low flow rate.

### 2.4 Flow cytometry

THP-1 cells (1×10^6^ cells/well) were seeded in 12-well plates. After treatment, cells were exposed to 10 mM diacetyldichlorofluorescein (Beyotime, China) and 50 nM MitoSOX (MCE, China) for 30 minutes at room temperature, and washed twice with PBS. Cell surface markers were determined by direct staining with PerCP anti-human CD14 antibodies (301847, Biolegend) following the manufacturer’s protocol. ROS, mitoROS and CD14 levels were assessed using flow cytometry (Beckman, CytoFLEX, USA) and analyzed using FlowJo software (Version 10.8.1).

### 2.5 Quantitative polymerase chain reaction

Total RNA was extracted from cells using TRIzol reagent (Invitrogen, USA), following the manufacturer’s instructions. Subsequently, the extracted RNA was reverse-transcribed into cDNA using Hifair III 1st strand cDNA Synthesis SuperMix (Yeasen, China). The relative expression levels of various target genes were quantified by q-PCR employing Hieff UNICONT^®^ Universal Blue qPCR SYBR Master Mix (Yeasen, China). For normalization, gene expression was referenced to ACTB. The primer sequences for qPCR are listed in **Supplementary Table 2**.

### 2.6 Western blot

Approximately 20 µg of protein were separated by 10% sodium dodecyl sulfate-polyacrylamide gel electrophoresis (SDS-PAGE) and subsequently transferred to polyvinylidene fluoride membranes (Merck Millipore, USA). Following a 1-hour block in 5% bovine serum albumin (Yeasen, China), the membranes were incubated with primary antibodies overnight at 4°C and with secondary antibodies for 1 hour. β-actin was employed as a loading control. The bound antibodies were visualized using the enhanced chemiluminescence (ECL) western blotting detection system (Merck Millipore, USA). The information for primary antibodies used in the experiment is listed in **Supplementary Table 3**.

### 2.7 Immunofluorescence staining

After treatment, the cells were washed with pre-cooled PBS, then fixed with 4% paraformaldehyde for 10 minutes, and dropped on a slide to dry. Pap Pen circled cell location, PBS washed cells three times. The cells were further permeabilized with 0.3% Triton X-100 in PBS for 20 minutes, blocked with 3% BSA for 30 minutes at room temperature, then incubated with anti-pp65 (1:500) antibody at 4°C overnight, incubated with Alexa Fluor 488 secondary antibody (1:1000, Abcam) for 1 hour at room temperature. Nuclei were stained with DAPI. Finally, images were captured using a Leica X10 Confocal (Mannheim, German).

### 2.9 UHPLC-MRM-MS/MS analysis

THP-1 cells (6 × 10^6^ cells/well) were collected. Cells were frozen in liquid nitrogen and were thawed in 37°C water bath. Repeated the freeze-thaw cycle 3 times and stored at -80°C before use. UHPLC-MRM-MS/MS was performed by Shanghai Biotree Biotech Limited Company (China). The abundance of metabolites was measured and compared to internal standard controls according to protocols.

### 2.10 Cytokine detection

After treatment for 48 hours, the cell culture supernatant was collected to measure the release of tumor necrosis factor α (TNF-α), interleukin 6 (IL-6), and monocyte chemoattractant protein 1 (MCP-1/CCL2) and CXCL10. A simple plex assay, a multiplex biomarker immunoassay panel cartridge (ProteinSimple, Bio-Techne, SPCKE-PS-009400), was used to measure the content of these cytokines in the serum samples according to the manufacturer’s instructions. Briefly, the samples were removed from the freezer, thawed, diluted with sample buffer diluent at a 1:1 ratio, pipetted, mixed well, centrifuged to remove visible particles, added to the samples, and analyzed with the Ella platform (ProteinSimple, Bio-Techne).

### 2.11 ELISA and glutathione detection

The LPS, vitamin B12 level, and glutathione/oxidized glutathione (GSH/GSSG) content were measured using the LPS (E09945h, CUSABIO), vitamin B12 ELISA kit (E07903h, CUSABIO) and GSH and GSSG (S0053, Beyotime), following the manufacturer’s instructions. After treatment, the cell culture supernatant and cells were separated by centrifugation at 1000g for 5 minutes. The cells were washed once with cold PBS and diluted to a concentration of 100 million/ml, after which the cells and supernatant were placed at -20°C overnight and detected the vitamin B12 and GSH/GSSG radio according to manufacturer’s protocol.

### 2.12 RNA-seq data collection and correlation analysis

Microarray datasets (GSE72509 and GSE112087) and the SLE patient monocyte RNA-seq dataset (GSE164457) were collected from the Gene Expression Omnibus database. The large-scale bulk transcriptome study of 27 immune cell types for SLE was obtained from the National Bioscience Database Center Human Database (Dataset ID: JGAS000486) (21). Microarray datasets were integrated and subjected to differential expression analysis using the limma package. Correlation analysis of TCN2 expression with clinical symptoms, clinical test results, and TLR4 expression, as well as ISGs score, were performed using the ggstatsplot and BayesFactor packages in the R environment.

### 2.13 Statistical analysis

Experimental results are expressed as mean ± SD and were subjected to unpaired Student’s t-test, one-way ANOVA and (or) two-way ANOVA, followed by Tukey’s or Dunnett’s multiple comparisons posttest. All analyses of experimental data were executed using GraphPad Prism 9 software (San Diego, USA). Statistical significance was established at *p* < 0.05.

## 3 Results

### 3.1 Monocytes from SLE patients exhibit elevated TCN2 expression

**Figure 1:**
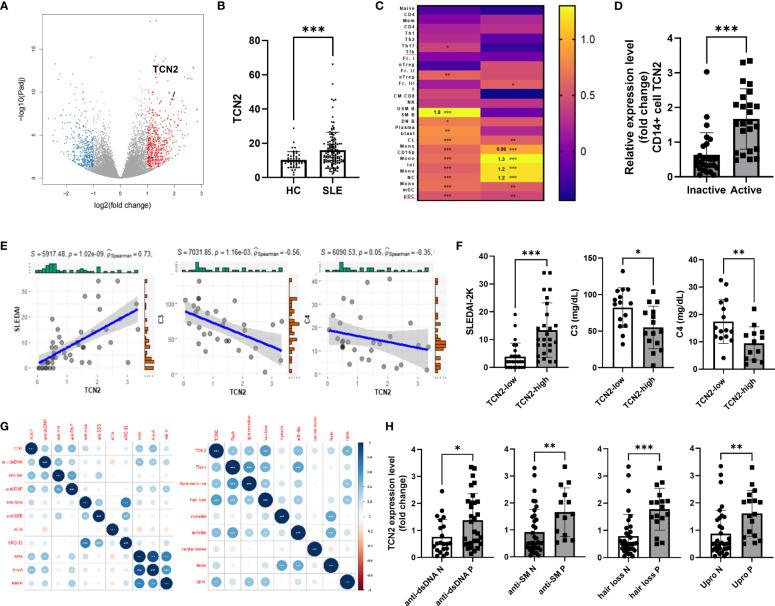
Increased TCN2 expression in monocytes from SLE patients associated with disease progression and tissue damage. **(A)** Volcano plot of DEGs between HC and SLE patients (HC=10, SLE patients=9). **(B)** TCN2 expression in HC and SLE patients (GSE72509 and GSE112087). **(C)** Heatmap of TCN2 transcript in immune cells from HC vs. inactive SLE patients (left) and inactive vs. active SLE patients (right). **(D)** qPCR analysis of TCN2 mRNA expression in monocytes from inactive and active SLE patients (inactive SLE patients=27, active SLE patients=24). **(E)** Linear regression analysis of TCN2 expression and SLEDAI-2K scores (n=51), C3, and C4 (n=35). **(F)** SLEDAI-2K scores, C3, and C4 levels of SLE patients with high and low TCN2 expression levels. **(G)** Correlation analysis of TCN2 expression and clinical symptoms and autoantibodies (n=51). **(H)** TCN2 expression levels in autoantibody-positive or negative groups in SLE patients. Data are mean ± SD. *P < 0.05, **P < 0.01, and ***P < 0.001 according to an unpaired t-test. HC, healthy control; Anti-dsDNA, Anti-double-stranded DNA; anti-SM, anti-Smith; anti-RNP, anti-ribonucleoprotein; anti-SSA/B, anti-Sjogren syndrome A/B; anti-ACA, anti-centromere antibody; ARO.52, anti-RO.52; AHA, anti-histone antibodies; AnuA, anti-nucleosome antibody; RIP.P, Anti-ribosomal P protein.

We identified elevated TCN2 expression in individuals with SLE through the analysis of public available microarray datasets (GSE72509 and GSE112087) and our RNA-seq analysis (**Figures 1A, B**) (22), while vitamin B12 transporter TCN1 of the same family was not (**Supplementary Figure 2**) (16). Subsequently, qPCR results confirmed the upregulation of TCN2 in PBMC from SLE patients compared to healthy control (HC, **Supplementary Figure 3A**). To pinpoint the cells responsible for TCN2 expression changes in PBMC, we conducted a search using a large-scale bulk transcriptome study of 27 immune cell types for SLE (21). The results indicated a significant elevation of TCN2 expression in non-transforming memory B cells from inactive SLE patients compared to healthy individuals. More importantly, TCN2 expression in all types of monocytes was notably increased in patients with high disease activity compared to those with inactive SLE (**Figure 1C**), implying the potential involvement of TCN2 in SLE progression through the regulation of monocyte function.

To clarify changes in TCN2 expression in monocytes of SLE patients, we isolated monocytes from both SLE patients and HC. We confirmed that TCN2 was significantly upregulated in SLE patients, particularly in those with active SLE (**Figure 1D**, **Supplementary Figure 3B**). We then investigated the correlation between monocyte TCN2 expression and disease progression as well as tissue damage. The expression of TCN2 in monocytes of SLE patients showed a positive correlation with SLEDAI-2K score, and a negative association with complement C3 and C4 levels (**Figure 1E**). Patients were divided into high and low groups based on TCN2 expression, and the high group exhibited higher SLEDAI-2K scores and lower complement C3 and C4 levels (**Figure 1F**). In addition, TCN2 expression was found to be associated with certain symptoms such as alopecia, elevated urinary proteins (>0.5 g/24 hours), arthritis, skin rash, light sensitivity, and various autoantibodies including anti-dsDNA, SM, RNP, histone, and nucleosome antibody (**Figure 1G**). Patients exhibiting these symptoms or autoantibodies displayed higher TCN2 expression in monocytes (**Figure 1H**, **Supplementary Figure 3C**). Taken together, monocytes from patients with SLE exhibit elevated expression of TCN2, which is associated with disease activity and involvement of the skin, kidneys, and joints.

### 3.2 Activated TLR4 increase TCN2 expression in monocytes

**Figure 2:**
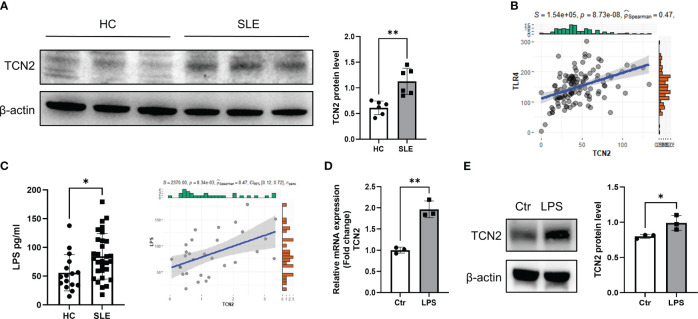
Elevated LPS in the blood of SLE patients promotes elevation of TCN2 in monocytes. **(A)** TCN2 expression in healthy human monocytes treated with SLE patient or HC serum for 24 hours (n=6). **(B)** Correlation analysis of TLR4 and TCN2 expression in monocytes. **(C)** LPS levels in blood of SLE and HC (HC=16, SLE=30) detected by ELISA. **(D)** Correlation analysis between LPS levels in the blood of SLE patients and TCN2 expression in monocytes of patients (SLE=30). **(E)** After 24 hours of 100 ng/ml LPS treatment, TCN2 expression in healthy human monocytes measured by western blot (n=3). Data are mean ± SD. *P < 0.05 and **P < 0.01 according to an unpaired t-test. HC, healthy control.

To investigate the reasons for the upregulation of TCN2 expression, CD14+ monocytes were isolated from HC and treated with serum from patients with active SLE. Increased TCN2 expression was observed after treatment with serum from SLE patients (**Figure 2A**).

LPS is a classical TLR4 agonist that has been reported increased in SLE patients (23, 24). Monocytes are one of the major cells responsible for TLR4-mediated inflammatory responses (3, 25). Elevated LPS may promote TCN2 expression in monocytes. We analyzed the correlation between TCN2 and TLR4 in the transcriptome of SLE patients and found a positive association between TCN2 and TLR4 levels (**Figure 2B**). Additionally, LPS level was elevated in the serum of SLE patients and was positively correlated with TCN2 expression in monocytes (*P*=8.34e-03, R^2^ = 0.47), as determined by ELISA and correlation analysis (**Figure 2C**). Furthermore, the mRNA and protein level of TCN2 increased in monocytes after direct treatment with LPS (**Figures 2D, E**). These results confirmed that increased LPS induces monocytes to upregulate TCN2 expression in SLE patients.

### 3.3 Heterozygous knockout TCN2 inhibits THP1 cell proliferation by G2/M phase arrest

**Figure 3:**
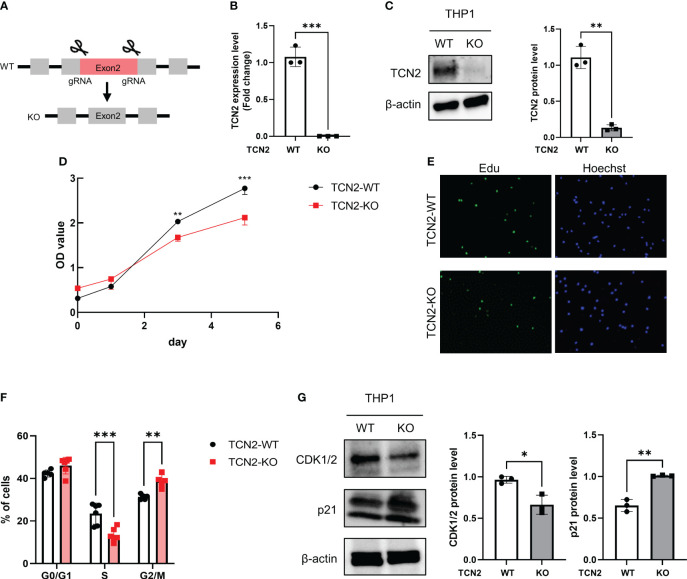
TCN2 knockdown in THP1 monocytes line inhibits cell cycle progression. **(A)** TCN2 knockdown strategy. **(B)** TCN2 knockout fragment detected by qPCR. **(C)** Western blot analysis of knockdown efficiency in TCN2-KO cells. **(D)** CCK-8 assay of TCN2-WT and TCN2-KO cells from 0 to 5 days. **(E)** EdU incorporation assay and **(F)** cell cycle analysis of TCN2-WT and TCN2-KO cells. **(G)** Western blot analysis of G2 phase regulator CDK1/2 and p21 in TCN2-WT and TCN2-KO cells. Data are mean ± SD (n = 3). *P < 0.05, **P < 0.01, and ***P < 0.001 according to an unpaired t-test. TCN2-KO, TCN2 knockout.

To explore the role of TCN2 *in vitro*, heterozygous knockout TCN2 THP1 cell was constructed (**Figure 3A**). The qPCR and western blot analysis confirmed successful downregulation of TCN2 in THP1 cells (**Figures 3B, C**).

The CCK-8 assay indicated that TCN2-KO cells exhibited slower proliferation compared to wild-type (TCN2-WT) cells (**Figure 3D**). The heterozygous knockout of TCN2 resulted in reduced DNA replication (**Figure 3E**) and G2/M phase arrest (**Figure 3F**). This suggests that cells carry out DNA replication and repair during the mitosis phase. Furthermore, western blot analysis indicated that TCN2-KO cells exhibited reduced expression of the checkpoint protein CDK1/2 and increased expression of the suppressor protein p21 compared to TCN2-WT cells (**Figure 3G**).

### 3.4 Heterozygous knockout TCN2 alleviates LPS-induced inflammatory response

**Figure 4:**
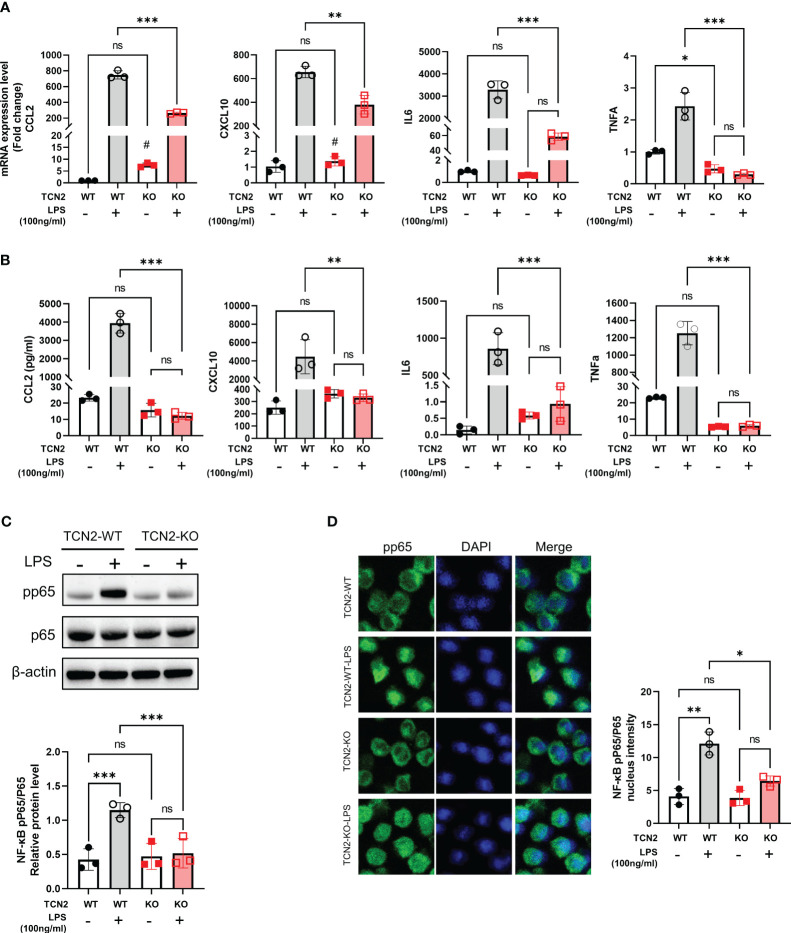
TCN2 knockdown alleviates LPS-induced inflammatory response. **(A)** qPCR analysis (24 hours) and **(B)** Ella analysis (48 hours) of CCL2, CXCL10, IL6, and TNFα in THP1 cells with or without 100 ng/ml LPS treatment after TCN2 knockdown. Data are mean ± SD (n=3). **(C)** pp65 and p65 in THP1 cells with or without LPS treatment for 60 min accessed by western blot. **(D)** Immunofluorescence staining of pp65 in THP1 cells with or without 100 ng/ml LPS treatment for 30 min. Data are mean ± SD (n = 3). #*P* < 0.05, indicating before LPS treatment compared with after LPS treatment in TCN2-KO cells, *P < 0.05, **P < 0.01, ***P < 0.001, and ns indicate non-significance according to two-way ANOVA.

We further evaluated the impact of TCN2 heterozygous knockout on cytokines and proinflammatory factors related to disease progression. As depicted in **Figure 4A**, TNFA and IL6 mRNA increases were not induced in TCN2-KO cells after LPS treatment. Although LPS induced the upregulation of CCL2 and CXCL10 expression in TCN2-KO cells, their expression levels were significantly lower than those in LPS-induced TCN2-WT cells. More importantly, Ella results confirmed that TCN2-KO reduced the release of CCL2, CXCL10, IL6, and TNFα induced by LPS. The levels of these cytokines after LPS stimulation were not significantly different from those before treatment (**Figure 4B**).

Furthermore, TCN2 heterozygous knockout significantly reduced the phosphorylation of nuclear factor kappa B (NF-κB) p65, as well as the nuclear translocation of pp65 under LPS stimuli (**Figures 4C, D**). These results suggested that TCN2 heterozygous knockout alleviates LPS-induced inflammatory response.

### 3.5 TCN2 dysfunction hinders folate one-carbon metabolism

**Figure 5:**
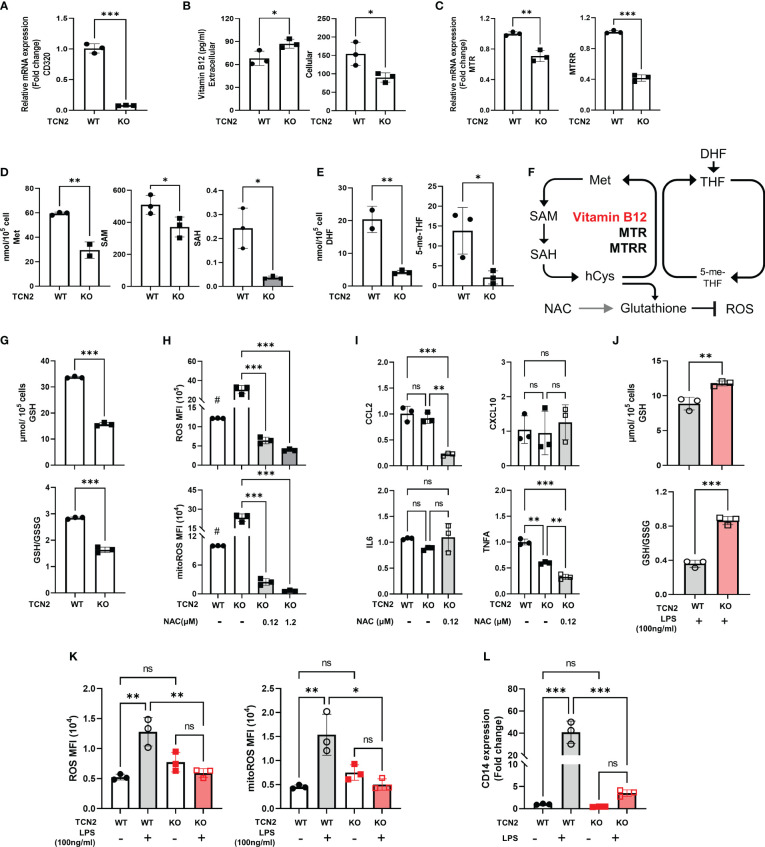
Knockdown of TCN2 blocks folate one-carbon metabolism. Change in **(A)** mRNA expression of CD230, **(B)** vitamin B12 levels, **(C)** mRNA expression of TCN2 coworker (MTR and MTRR), and **(D)** metabolites level from methionine cycle, and **(E)** folate cycle after TCN2 dysfunction. **(F)** Diagram of folic acid one-carbon metabolism. **(G)** GSH and GSH/GSSG ratio in TCN2-WT and TCN2-KO cells. **(H)** ROS and mitoROS and **(I)** inflammatory factors in TCN2-KO cells with or without NAC treatment for 24 hours. **(J)** GSH and GSH/GSSG ratio in TCN2-WT and TCN2-KO cells after LPS treatment for 24 hours. **(K)** ROS, mitoROS, and **(L)** CD14 in TCN2-KO cells and TCN2-WT cells were treated with or without LPS for 24 hours. Data are mean ± SD (n = 3). # *P* < 0.05, indicates TCN2-KO cell without NAC treatment compared to TCN2-WT cell without NAC treatment, *P < 0.05, **P < 0.01, ***P < 0.001, and ns indicate non-significance according to unpaired t-test **(A–G, J)**, two-way ANOVA **(H, I, K, L)**. GSH, Glutathione; GSSG, oxidized glutathione; MTR, methionine synthase; MTRR, methionine synthase reductase; Met, methionine; SAM, S-adenosylmethionine; SAH, S-adenosyl-l-homocysteine; DHF, dihydrofolate; 5-me-THF, 5-methyltetrahydrofolic acid.

In most cells of the human body, vitamin B12 binds to TCN2 and mediates endocytic uptake through CD320 (26). We subsequently assessed vitamin B12 levels and the transfer factor CD320 in TCN2 KO cells. As illustrated in **Figures 5A** and **B**, heterozygous TCN2 knockout resulted in a decreased cellular vitamin B12 and CD320 expression, indicating that vitamin B12 absorption was inhibited. In addition, MTR and MTRR were downregulation in TCN2-KO cells, implying an inhibition in conversion of homocysteine to methionine (**Figure 5C**). We then assessed changes in catalytic enzyme and metabolite levels in one-carbon metabolism. As expected, the qPCR analysis identified a significant downregulation of enzymes involved in the folate cycle and methionine cycle after TCN2 dysfunction (**Supplementary Figure 4**). Consistently, metabolites from the one-carbon pathway were lower in TCN2-KO cells than in TCN2-WT cells (**Figures 5D, E**), suggesting impaired folate one-carbon metabolism (**Figure 5F**).

GSH is an essential downstream metabolite of the methionine cycle which functions as an antioxidant (27). We evaluated the alterations in GSH and ROS after TCN2 knockout. We found a recognizable decrease in GSH and GSH/GSSG levels, coupled with an increased ROS and mitoROS, but no significant increase in inflammatory factors expression (CCL2, CXCL10, IL-6, and TNFA) (**Figures 5G–I**), suggesting that a slight increase in ROS does not trigger the release of inflammatory factors in TCN2-KO cells. Furthermore, the addition of NAC, a precursor to GSH, resulted in a significantly reduced ROS, mitoROS, and expression of CCL2 and TNFA in TCN2-KO cells (**Figures 5H, I**). LPS can induce massive ROS production (28). Importantly, GSH levels, GSH/GSSG ratios, and ROS remained stable after LPS treatment in TCN2-KO cells (**Figures 5J, K**), suggesting that TCN2 knockdown prevented reactive ROS elevation and GSH depletion. CD14 acts as a co-receptor for LPS and regulates cellular TLR4 signaling. In line with this, TCN2-KO cells did not exhibit a noticeable increase in CD14 expression under LPS stimuli, whereas TCN2-WT cells showed a significant increase (**Figure 5L**).

### 3.6 TCN2 promotes THP1 cell proliferation and CCL2 release by regulating methionine cycle

**Figure 6:**
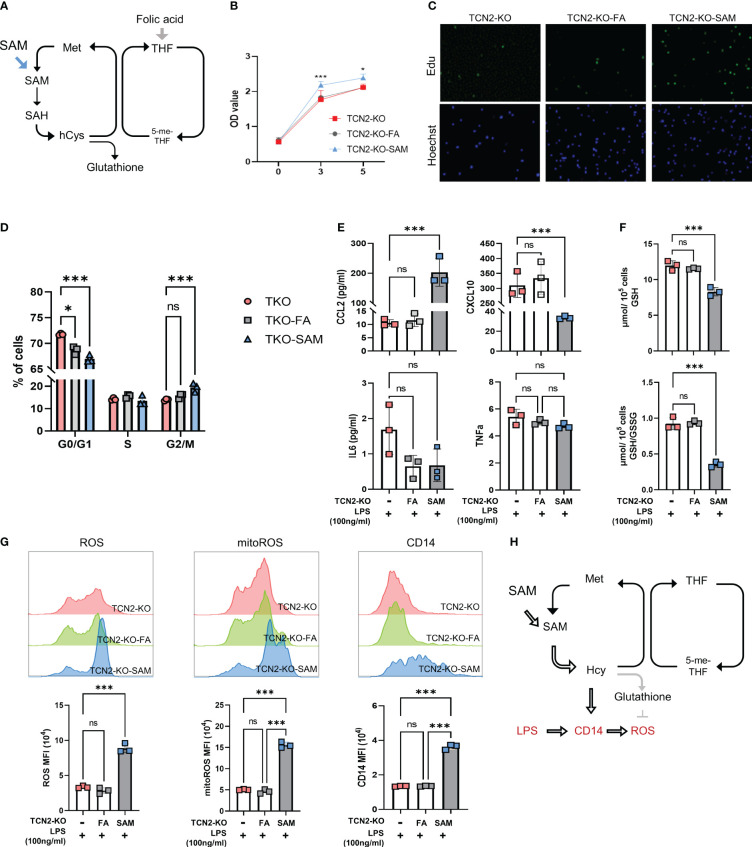
One-carbon metabolism supporter SAM hinders TCN2-mediated inhibition of cell proliferation and inflammatory responses rather than folic acid. **(A)** FA and SAM support one-carbon metabolism. TCN2-KO cells and TCN2-WT cells were treated with 1 μg/ml FA or 10 μM SAM for 2 days, and cell proliferation in TCN2-KO cells was evaluated by **(B)** CCK-8 assay, **(C)** EdU incorporation assay, and **(D)** Cell cycle detection. TCN2-KO cells and TCN2-WT cells were pretreated with 1 μg/ml FA or 10 μM SAM for 1 day, and then treated with 100ng/ml LPS for 24 hours. **(E)** The release of CCL2, CXCL10, IL6, and TNFα were detected by ELISA; **(F)** GSH and GSH/GSSG levels were detected by GSH detection kit; **(G)** CD14, ROS, and mitoROS were measured by flow cytometry. **(H)** Schematic diagram of SAM hampering TCN2-mediated inflammatory response. Data are mean ± SD (n=3). *P < 0.05, ***P < 0.001, and ns indicate non-significance according to one-way ANOVA. FA, folic acid; SAM, S-adenosyl-methionine; GSH, glutathione; GSSG, oxidized glutathione.

Folate one-carbon metabolism includes the folate cycle and the methionine cycle. To investigate whether TCN2 regulates monocyte function by affecting folate one-carbon metabolism, we treated TCN2-KO cells with the support of the folate cycle (FA) or the methionine cycle (SAM) (**Figure 6A**). FA enters cells through its transporters (29). We first examined whether TCN2 KO affects FA transport. After TCN2 inhibition, the folate cycle is inhibited, which reduces the cell’s demand for FA and downregulates the folate transporter (FORLR2 and SLC19A1). However, they increased to normal levels after FA supplementation (**Supplementary Figure 5**), suggesting that folate absorption was less affected by TCN2 knockout.

Subsequently, we assessed cell proliferation and found SAM treatment reduced the G2/M phase rate and promoted cell proliferation in TCN2-KO cells. In contrast, FA treatment did not significantly affect the proliferation of TCN2-KO cells (**Figures 6B–D**). These findings suggested that TCN2 knockout suppresses cell proliferation by inhibiting the methionine cycle. Furthermore, we detected the release of inflammatory factors induced by LPS. **Figure 6E** showed an increase in CCL2 secretion induced by LPS after SAM supplementation, while CXCL10 expression was further inhibited. IL6 and TNF levels remained unchanged. The release of CCL2 is dependent on ROS (30, 31). As expected, TCN2-KO cells with SAM pretreatment showed a significant increase in LPS-induced ROS and GSH consumption, coupled with upregulation of CD14 (**Figures 6F–G**). Consistently, Hcy inhibited TCN2-mediated suppression of ROS and mitochondrial ROS, while increasing CD14 (**Supplementary Figure 6**). Furthermore, the changes in inflammatory factors after FA supplementation were not significantly different from those in the control group. These findings suggested that TCN2 enhances LPS-induced CCL2 release by regulating the methionine cycle rather than the folate cycle (**Figure 6H**).

### 3.7 Folic acid deficiency exacerbates inflammatory response in THP1 cells

**Figure 7:**
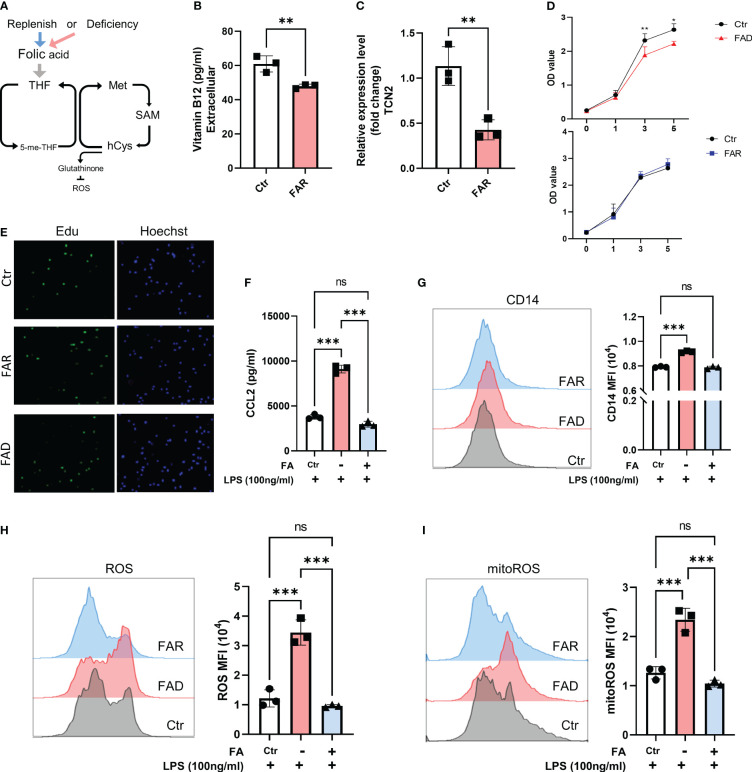
Folic acid deficiency inhibits cell proliferation but enhances THP1 cell inflammatory response. **(A)** THP1 cells were folic acid deficient or supplemented for 3 days, and **(B)** cellular vitamin B12 levels were assessed by ELISA, **(C)** and TCN2 expression in THP1 cells was detected by qPCR. **(D)** Cell proliferation was evaluated by CCK-8 assay and **(E)** EdU incorporation assay. **(F)** ELISA analysis of CCL2 in THP1 cell under LPS stimuli for 1 day after folic acid deficiency or supplement for 3 days. **(G)** CD14, **(H)** ROS, and mitoROS were measured by flow cytometry. Data are mean ± SD (n=3). *P < 0.05, **P < 0.01, ***P < 0.001, and ns indicate non-significance according to unpaired t-test **(B–D)** or one-way ANOVA **(F–I)**.

Folate has been implicated in the inflammatory response (32). We investigated whether folate deficiency could mimic the effects of TCN2 on monocyte cells by depleting THP1 cells of folate using FA-free medium (**Figure 7A**).

As anticipated, folate depletion resulted in decreased cellular expression of vitamin B12 and TCN2 (**Figures 7B, C**). In line with previous research (9), FA depletion caused cell growth retardation, while FA supplementation did not significantly affect cell proliferation (**Figures 7D, E**, **Supplementary Figures 7A, B**). However, folate deficiency induced a stronger inflammatory response, as evidenced by elevated CD14 expression and increased release of CCL2 and TNFα in cells with low folate levels compared to those with normal levels. Additionally, pretreatment with FA did not result in a reduced response to LPS stimulation (**Figures 7F, G**, **Supplementary Figure 7C**). Moreover, folate-deficient cells exhibited a significant increase in ROS and mitoROS compared to cells with folate supplements and the normal folate group under LPS stimuli (**Figures 7H, I**). These findings suggest that the cellular response under FA deficiency differs from the impact of TCN2 inhibition, emphasizing the intricate role of TCN2 in modulating cellular responses.

## 4 Discussion

Accumulating evidence has indicated the important role of monocytes in SLE (25, 33). In this study, we have demonstrated that the vitamin B12 transporter TCN2 is overexpressed in monocytes from SLE patients. The elevated level of TCN2 is positively correlated with the severity of the disease and is associated with manifestations such as arthritis, kidney injury, and hair loss. In addition, the heterozygous knockout of TCN2 impaired THP1 cell proliferation by retarding cell cycle progression and attenuated the release of CCL2 induced by LPS. These effects were rescued by SAM and Hcy, metabolites in methionine cycle, while FA treatment proved to be ineffective. Notably, although folate deficiency inhibited monocyte proliferation, it failed to replicate the impact of TCN2 downregulation on the THP1 inflammatory response.

Transcobalamin is responsible for transporting vitamin B12 from the blood into cells, including TCN1 and TCN2 which are widely distributed in cells. TCN2 is mainly expressed in monocytes, whereas TCN1 is mainly expressed in basophils. TCN2 has higher specificity and turnover rate compared with TCN1 and is considered the best biomarker of active and available B12 (16). Patients with SLE exhibit lower levels of vitamin B12 and abnormal fluctuations in TCN2 levels (17–19). In particular, patients with active disease release large amounts of TCN2 in a short time (17). Consistent with previous studies, we found a significant upregulation of TCN2 in SLE patients with high disease activity. TCN2 expression in monocytes was correlated with markers of disease progression, including SLEDAI-2K and complement C3/C4, suggesting that TCN2 could serve as a potential biomarker for SLE progression. Besides, the reduction of C3 and C4 impaired the ability of macrophages to phagocytose apoptotic and necrotic cells, exacerbating the autoimmune response (34). It was implied that TCN2 may have an impact on macrophage function.

One-carbon metabolism is a crucial metabolic pathway for the biosynthesis of nucleotides and methyl group donors. Nucleotides are essential for DNA replication, DNA repair, gene expression, and protein translation via ribosomal RNA. Methyl groups are used for methylation of proteins, RNA, and DNA (35). Thus, folate one-carbon metabolism is essential for cell proliferation (7, 12). Studies have shown that mitochondrial enzymes involved in the one-carbon pathway, namely SHMT2, MTHFD2, and MTHFD1L, affect the proliferation rate of tumor cells (8, 36, 37). Ron-Harel N. et al. reported that the salvage pathway of serine supply and mitochondrial one-carbon metabolism affects T cell proliferation and survival (Ron-Harel N, et al. Cell Metab. 2016). This study showed that TCN2 knockout inhibits vitamin B12 absorption and leads to a block in one-carbon metabolism. The levels of vitamin B12 and its transporter CD320 were reduced in TCN2-KO cells, indicating inhibited vitamin B12 absorption. Furthermore, the expression levels of MTR and MTRR were downregulated in TCN2-KO cells. It is speculated that the absence of vitamin B12 leads to the inhibition in the conversion of homocysteine to methionine, which reduces the cell’s demand for MTR and MTRR, resulting in the down-regulation of MTR and MTRR gene expression. UHPLC-MRM-MS/MS analysis confirmed a decrease in one-carbon metabolites after TCN2 dysfunction, indicating one-carbon metabolism was inhibited in TCN2-KO cells. Consistent with previous studies, inhibited one-carbon metabolism resulted in cell cycle arrest in the G2/M phase, characterized by an increase in p21 levels and a decrease in CDK1/2 expression after TCN2 KO. It has been suggested that cells carry out DNA replication and repair during the G2/M phase.

In additon, dysfunction of TCN2 limited the expression and release of inflammatory cytokines induced by LPS, such as CCL2, CXCL10, IL6, and TNF-α. These inflammatory markers also correlate with SLEDAI-2K, levels of dsDNA (CCL2, CXCL10), the presence of lupus nephritis (CCL2, CXCL10, and TNF-α), and arthritis (IL6) (38, 39). Among these inflammatory factors, CCL2 is widely regarded as a biomarker of SLE kidney injury and treatment response (40). Our results suggested that TCN2 knockout modulates SAM and Hcy in the methionine cycle, leading to the inhibition of LPS-induced CCL2 release. LPS is a classical TLR4 agonist and is considered a risk factor for SLE (41). Intestinal LPS biosynthesis is enhanced in SLE patients and mouse models, inducing proinflammatory activity (23, 24). One-carbon metabolism blockade was found to reduce macrophage LPS responsiveness by impairing the expression of TLR4 co-factor, CD14. In line with this, we also found that CD14 was not significantly altered in TCN2-KO cells under LPS stimulation (28). CD14 facilitates TLR4 dimerization by transferring LPS to MD2, forming TLR4-MD2 heterodimers (42). Excessive TLR4 activation has been shown to enhance cell death and systemic inflammatory responses (43). Our results revealed that TCN2 dysfunction dampened TLR4 activation and downstream ROS/NF-κB pathways by restricting CD14 expression under LPS stimuli. Pretreatment with SAM and Hcy successfully reversed the alterations in CD14 expression induced by LPS and CCL2 release induced by ROS in TCN2-KO cells. This underscores the pivotal role of methionine metabolism in TCN2’s effects on monocytes. It is worth noting that SAM and its associated methylation reactions have been reported to coordinately modulate histone methylation modifications, promoting inflammation and driving inflammatory macrophages. Furthermore, it has been reported that methionine deprivation can lead to an overall decrease in histone methylation (44, 45). Therefore, overexpression of TCN2 may also regulate inflammatory responses by affecting histone methylation. Further research is needed to clarify the impact of TCN2 or B12 on methylation in inflammation.

The methionine cycle can affect cellular oxidative stress by influencing the production of the antioxidant GSH. It is a tripeptide that contains cysteine, glutamic acid, and glycine residues. Specifically, Hcy is synthesized with serine to form cystathionine, which is then cleaved and hydrolyzed to cysteine. Cysteine then combines with glutamic acid and glycine to synthesize GSH (46, 47). We observed decreased GSH and increased ROS in TCN2-KO cell without altering CCL2, CXCL10, IL6, and TNFA levels, suggesting that increased ROS is not high enough to induce the release of inflammatory factors in TCN2-KO cells. However, under LPS stimulated, TCN2 knockdown blunted LPS-induced TLR4 activation under LPS stimulation and resulted in unchanged ROS and CCL2 release.

Although the folate cycle is a crucial component of the one-carbon cycle, supplementation with FA did not alleviate cell cycle arrest or mitigate inflammatory responses in TCN2-KO cells. This may not be caused by folate malabsorption. FA absorption relies on folate carrier (RFC)- or thiamine carrier (PCFT)-mediated transport and folate receptor (FOLR)-mediated endocytosis (29). Folate transporter expression, including FOLR1, FORLR2, and SLC19A1, decreased after TCN2-KO but returned to normal levels after FA supplementation. A more reasonable explanation is that the folate cycle becomes largely uncoupled from the methionine cycle during LPS-induced inflammation (12). Cells do not rely solely on the folate cycle as the source of methyl groups in the methionine cycle under LPS stimuli. The serine synthesis (SSP) and pentose phosphate (PPP) pathways, which are offshoots of glycolysis, can provide one-carbon units for the methionine cycle through *de novo* ATP synthesis (12). Folate cycle, as one-carbon unit donor, is not always necessary for methylated Hcy. It also explains why folate deficiency exacerbates the inflammatory response in monocytes and does not produce similar effects as TCN2 knockdown (32). Since the SSP and PPP pathways can provide methyl groups to the methionine cycle, allowing for SAM production. However, Hcy cannot be converted into methionine by acquiring a methyl group, which cause homocysteine to accumulate (12). It has been reported that both SAM (48) and Hcy (49) have pro-inflammatory effects. Increased inflammation may be caused by Hcy accumulation.

Our study has some limitations. Anti-ds DNA antibodies can bind to and activate TLR4, and further research is needed to determine the effect of TCN2 on dsDNA antibodies stimulation (50, 51). It is a complex process to transport FA and SAM across the cell membrane. Neither molecule can enter cells by osmosis. Exogenous FA relies on a variety of transport enzymes to enter cells (29), while SAM enters cells through degradation, absorption, and resynthesis (52). As a result, cells may not absorb sufficient amounts of FA or SAM, leading to inadequate replenishment. Additionally, the reason for TCN2 knockout inhibiting LPS-induced CXCL10, IL6, and TNFA is unclear. Recent studies have reported that dysfunction of TCN2 resembles the effects of hypoxia, suggesting its impact on energy metabolism (53). Additionally, Vitb12 is an AhR antagonists (54). Interestingly, the functions of AhR and HIF-α, one of the major regulators of the hypoxia response, require binding to the aryl hydrocarbon receptor nuclear translocon (ARNT) to form a dimer (55), suggest that TCN2 may also act through both pathways. We will investigate TCN2 and vitb12 on inflammation and immune responses in further study.

In conclusion, our study revealed the distinct role of TCN2 in driving one-carbon flux on SLE-associated monocyte behavior. Elevated TCN2 levels may contribute to disease progression and tissue damage by promoting one-carbon flux, stimulating monocyte proliferation, and exacerbating TLR4-mediated inflammatory responses. Our findings suggest the role of B vitamins and one-carbon metabolism in monocytes from SLE patients. Inhibition of TCN2 may be a promising therapeutic approach to ameliorate SLE.

## Data availability statement

The datasets presented in this study can be found in online repositories. The names of the repository/repositories and accession number(s) can be found below: MTBLS9136 (Metabolights).

## Ethics statement

The studies involving humans were approved by the Human Ethics Committee of China-Japan friendship hospital. The studies were conducted in accordance with the local legislation and institutional requirements. The participants provided their written informed consent to participate in this study.

## Author contributions

BL: Conceptualization, Writing – original draft, Investigation. AL: Investigation, Writing – review & editing. YL: Investigation, Writing – review & editing. XZZ: Investigation, Writing – review & editing. JX: Funding acquisition, Writing – review & editing. XBZ: Funding acquisition, Writing – review & editing. KX: Funding acquisition, Writing – review & editing. YC: Funding acquisition, Writing – review & editing.
