# Supplementary material for: Transcobalamin 2 orchestrates monocyte proliferation and TLR4-driven inflammation in systemic lupus erythematosus via folate one-carbon metabolism
Source: Front Immunol. 2024 May 31;15:1339680. doi: 10.3389/fimmu.2024.1339680 (PMC11176449; doi:10.3389/fimmu.2024.1339680)
Supplement: Supplementary file 1 [file DataSheet_1.pdf]

Supplementary Figure

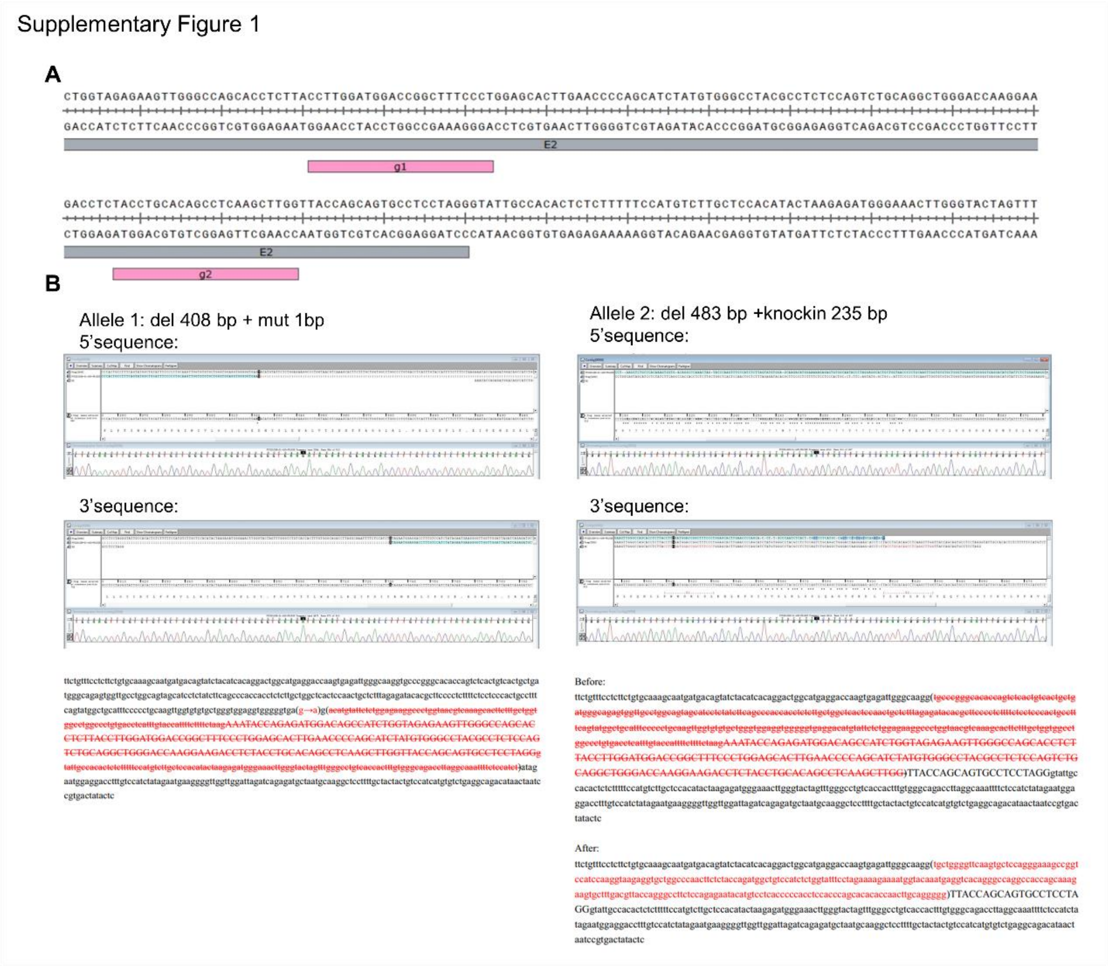

Supplementary Figure 1. Construction of TCN2 knockout THP1 cell line.

(A) Gene knockout strategy. **(B)** Sanger sequencing of TCN2-KO cells. Upper letters refer to the exon sequence while lower letters refer to the intron sequence. Red letters (crossed) refer to the deleted sequence. (g→a) means the original nucleotide is g and now has been mutated to a.

Supplementary Figure 2

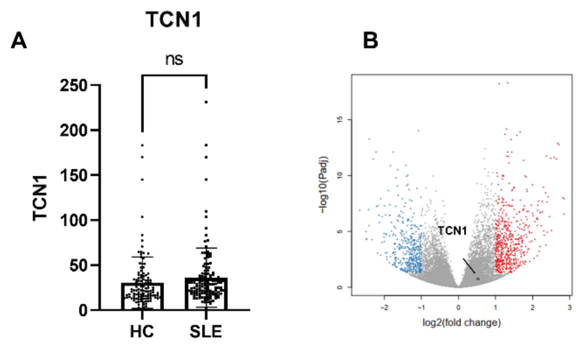

Supplementary Figure 2. TCN1 transcript in HC and SLE patients.

(A) TCN1 expression in HC and SLE patients (GSE72509 and GSE112087). **(B)** Volcano plot of DEGs between HC and SLE patients. ns indicates non-significance according to an unpaired t-test. HC, healthy control.

Supplementary Figure 3

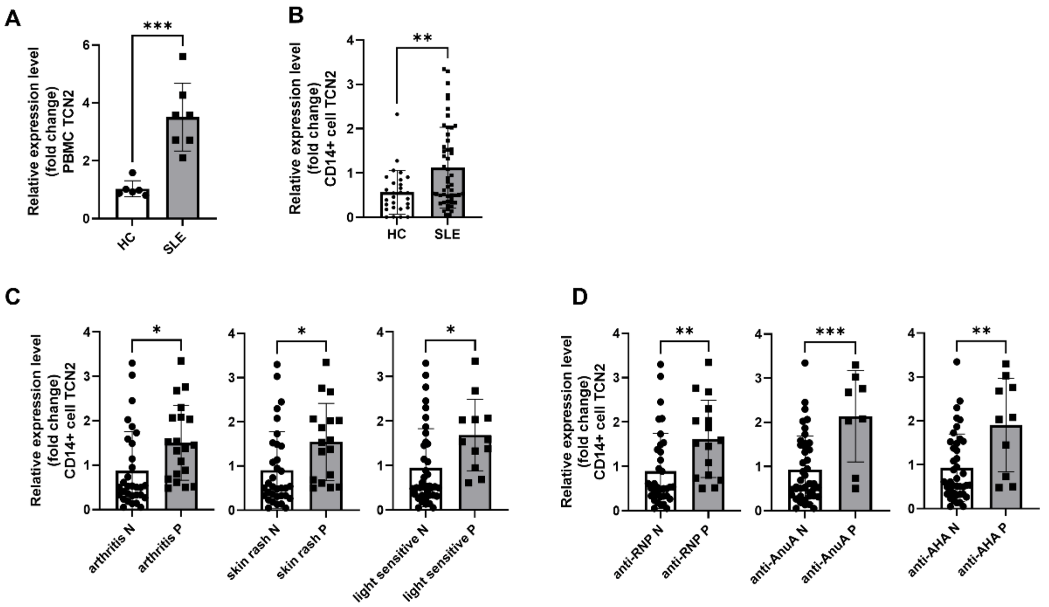

Supplementary Figure 3. Monocyte TCN2 expression in SLE patients correlates with disease progression

(A) qPCR analysis of TCN2 mRNA expression in PBMCs (HC=6, SLE patients=7) and **(B)** in monocytes from HC and SLE patients (HC=28, SLE patients=51). **(C)** TCN2 expression levels in clinical symptoms- and **(D)** autoantibody-positive or negative groups in SLE patients (SLE patients=51). **P* < 0.05, ***P* < 0.01, and ****P* < 0.001 according to an unpaired t-test. HC, healthy control. Anti-RNP, anti-ribonucleoprotein; anti-AHA, anti-histone antibodies; anti-AnuA, anti-nucleosome antibody.

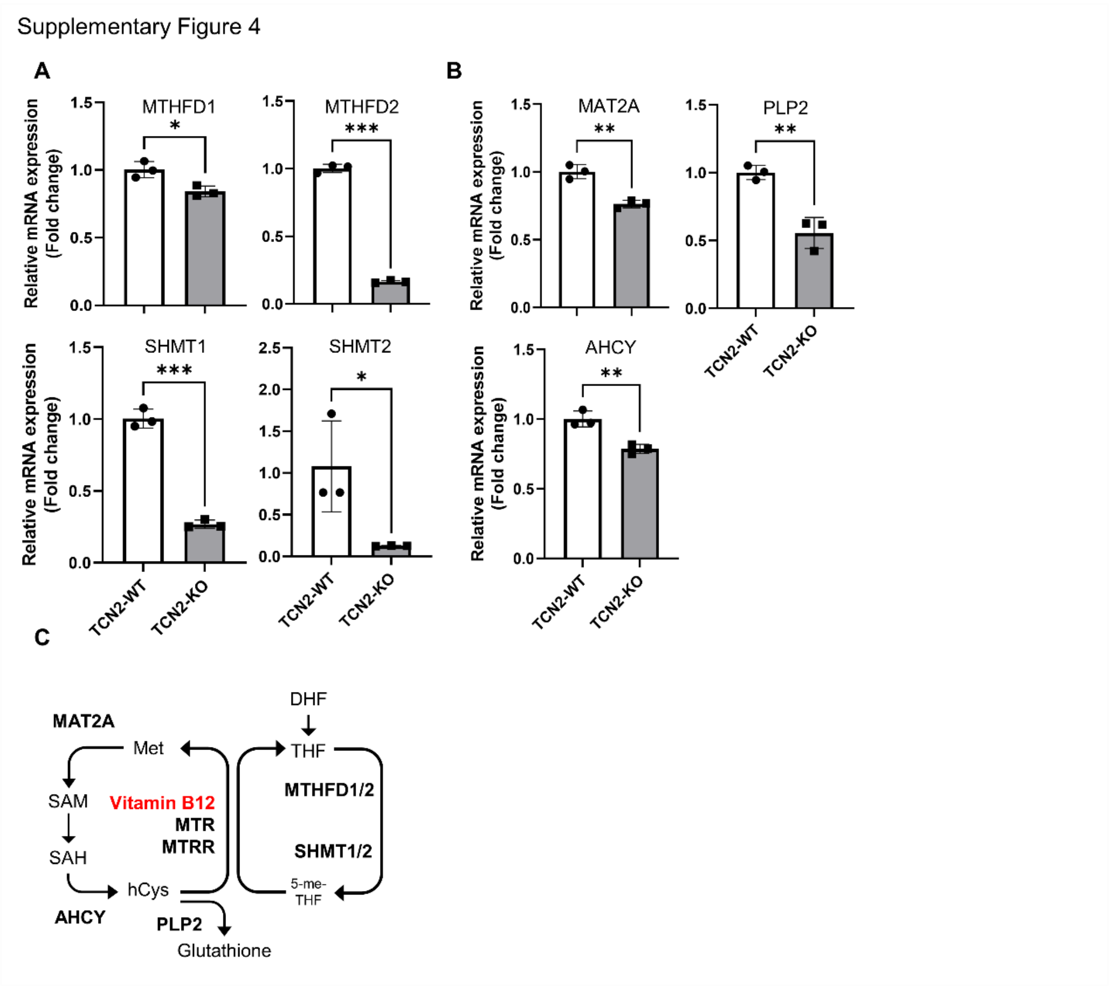

Supplementary Figure 4 Changes in the one-carbon pathway changes after TCN2 loss of function in THP1 cells.

(A) qPCR analysis of expression changes in key enzymes in folate or **(B)** methionine cycle after TCN2 dysfunction. **(C)** One-carbon metabolic pathway diagram. Data are

mean ± SD (n = 3). *P < 0.05, **P < 0.01, ***P < 0.001, and ns indicate non-significance according to t-test. MTHFD, methylenetetrahydrofolate dehydrogenase; SHMT, serine hydroxymethyltransferase; MAT2A, methionine adenosyltransferase 2A; PLP2, proteolipid protein 2; AHCY, adenosyl homocysteinase; MTR, methionine synthase; MTRR, methionine synthase reductase; Met, methionine; SAM, S-adenosylmethionine; SAH, S-adenosyl-l-homocysteine; DHF, dihydrofolate; 5-me-THF, 5-methyltetrahydrofolic acid.

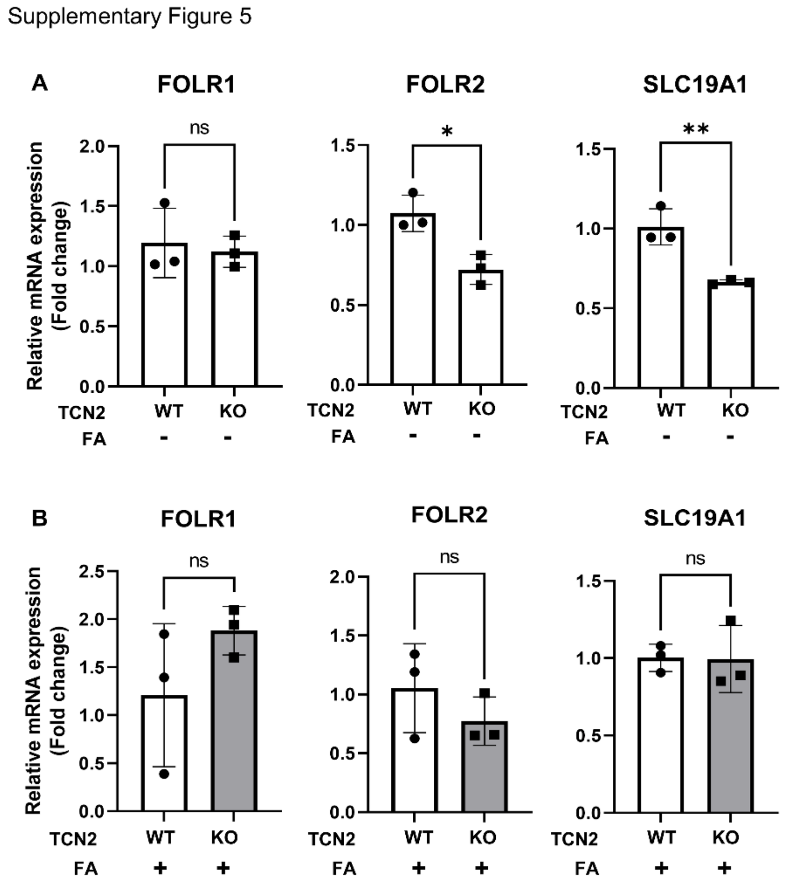

Supplementary Figure 5 Folate transporter alteration after TCN2-KO.

Folate transporter expression changes **(A)** before or **(B)** after FA treatment in TCN2-KO cells. Data are mean ± SD (n = 3). *P < 0.05, **P < 0.01, and ns indicate non-

significance according to t-test. FA, folic acid; FOLR, folate receptor; SLC19A1, solute carrier family 19 member 1.

Supplementary Figure 6

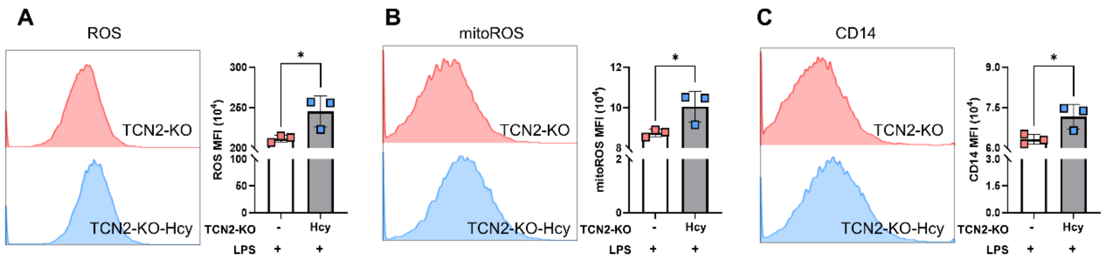

Supplementary Figure 6 Hcy hinders TCN2-mediated inhibition of ROS and CD14 increase induced by LPS.

(A) ROS, **(B)** mitoROS, and **(C)** CD14 level in TCN2-KO cells pretreated with 10μM Hcy for 1 day in the presence of 100ng/ml LPS for 24 hours. Data are mean ± SD (n = 3). **P* < 0.05 according to t-test. Hcy, homocysteine.

Supplementary Figure 7

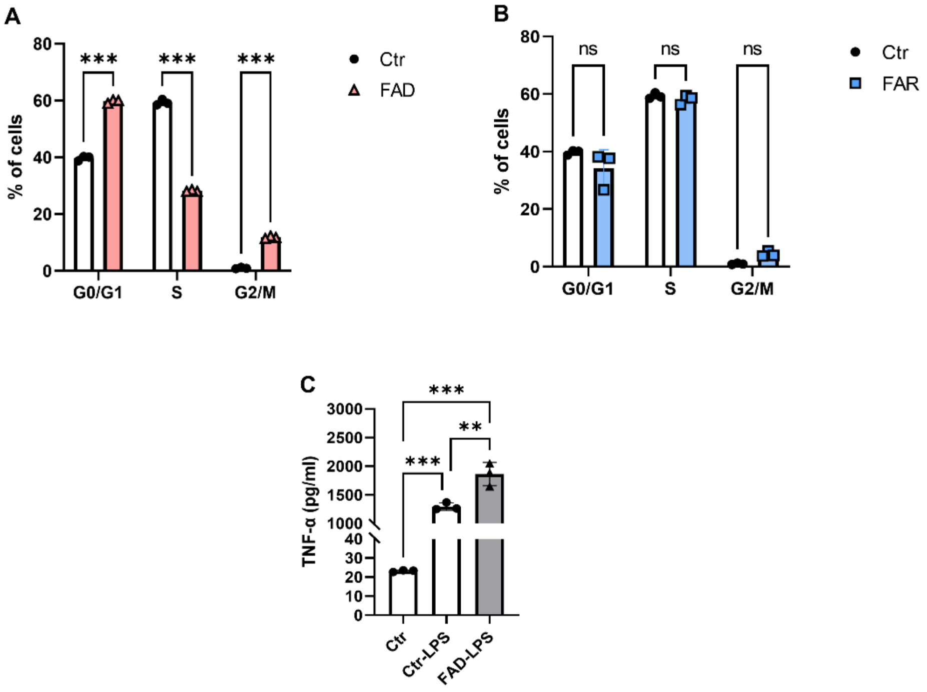

Supplementary Figure 7 Effects of folic acid changes on THP1 cell cycle and inflammatory response.

(A) Cell cycle analysis of THP1 cells after folate deficiency or **(B)** folate replenishment for 3 days. **(C)** Ella analysis of TNFα in THP1 cells with or without 100ng/ml LPS treatment for 24 hours. Data are mean ± SD (n=3). $^{**}P < 0.01$ and $^{***}P < 0.001$, and ns indicate non-significance according to (A-B) t-test, (C) one-way ANOVA, FAD, folate deficiency; FAR folate replenishment.
